# Immunohistochemical profiling of receptor tyrosine kinases, MED12, and TGF-βRII of surgically resected small cell lung cancer, and the potential of c-kit as a prognostic marker

**DOI:** 10.18632/oncotarget.14410

**Published:** 2016-12-31

**Authors:** Hiroshi Yokouchi, Hiroshi Nishihara, Toshiyuki Harada, Takashi Ishida, Shigeo Yamazaki, Hajime Kikuchi, Satoshi Oizumi, Hidetaka Uramoto, Fumihiro Tanaka, Masao Harada, Kenji Akie, Fumiko Sugaya, Yuka Fujita, Kei Takamura, Tetsuya Kojima, Mitsunori Higuchi, Osamu Honjo, Yoshinori Minami, Naomi Watanabe, Aya Goto, Hiroyuki Suzuki, Hirotoshi Dosaka-Akita, Hiroshi Isobe, Masaharu Nishimura, Mitsuru Munakata

**Affiliations:** ^1^ Department of Pulmonary Medicine, Fukushima Medical University, Fukushima, Japan; ^2^ Department of Translational Pathology, Hokkaido University Graduate School of Medicine, Sapporo, Japan; ^3^ Center for Respiratory Diseases, JCHO Hokkaido Hospital, Sapporo, Japan; ^4^ Clinical Oncology Center, Fukushima Medical University Hospital, Fukushima, Japan; ^5^ Department of Thoracic Surgery, Keiyukai Sapporo Hospital, Sapporo, Japan; ^6^ First Department of Medicine, Hokkaido University School of Medicine, Sapporo, Japan; ^7^ Department of Respiratory Medicine, National Hospital Organization Hokkaido Cancer Center, Sapporo, Japan; ^8^ Second Department of Surgery, University of Occupational and Environmental Health, Kita-kyushu, Japan; ^9^ Department of Thoracic Surgery, Kanazawa Medical University, Uchinada, Japan; ^10^ Department of Respiratory Disease, Sapporo City General Hospital, Sapporo, Japan; ^11^ Department of Respiratory Medicine, Teine Keijinkai Hospital, Sapporo, Japan; ^12^ Department of Respiratory Medicine, National Hospital Organization Asahikawa Medical Center, Asahikawa, Japan; ^13^ First Department of Medicine, Obihiro Kosei Hospital, Obihiro, Japan; ^14^ Department of Medical Oncology, KKR Sapporo Medical Center, Sapporo, Japan; ^15^ Department of Thoracic Surgery, Fukushima Red Cross Hospital, Fukushima, Japan; ^16^ Department of Thoracic Surgery, Fukushima Medical University, Fukushima, Japan; ^17^ Department of Respiratory Medicine, Sapporo-Kosei General Hospital, Sapporo, Japan; ^18^ Respiratory Center, Asahikawa Medical University, Asahikawa, Japan; ^19^ Department of Internal Medicine, Sunagawa City Medical Center, Sunagawa, Japan; ^20^ Center for Integrated Science and Humanities, Fukushima Medical University, Fukushima, Japan; ^21^ Department of Medical Oncology, Hokkaido University Graduate School of Medicine, Sapporo, Japan

**Keywords:** small-cell lung cancer, surgery, MED12, c-kit, immunohistochemistry

## Abstract

The limited number of available treatments for patients with small-cell lung cancer (SCLC) has prompted us to further investigate the biology of SCLC by molecular profiling. We collected formalin-fixed paraffin-embedded tumor samples from 127 patients with SCLC, who had undergone surgery at 16 institutions between January 2003 and January 2013, and analyzed the association between disease-specific survival and protein expression of c-kit, c-Met, epidermal growth factor receptor, human EGFR-related 2, vascular endothelial growth factor receptor II, anaplastic lymphoma kinase, mediator complex subunit 12 (MED12), and transforming growth factor beta receptor II (TGF-βRII) by immunohistochemistry (IHC). Of the 125 evaluable samples, all tumors expressed MED12, and 123 samples (98.4%) expressed TGF-βRII. MED12 was highly expressed in the nucleus in 92% of the positive samples while TGF-βRII was highly expressed in the cytoplasm in 55% of the positive samples. High c-kit expression was an independent favorable prognostic marker confirmed by multivariate analysis (hazard ratio: 0.543, 95% confidence interval: 0.310–0.953, *p* = 0.033). Both the relapse free-survival and overall survival of patients who underwent adjuvant chemotherapy were statistically longer in those with high c-kit expression (n = 38) than those with intermediate, low, or no c-kit expression (*n* = 19) (not reached *vs* 11.6 months, *p* = 0.021; not reached *vs* 25.9 months, *p* = 0.028). IHC for c-kit may offer a prognostic marker for early-stage SCLC, and the results for MED12 and TGF-βRII may suggest the biological characteristics of SCLC. Further investigation of the roles of their related molecules in early stage SCLC is required.

## INTRODUCTION

Small-cell lung cancer (SCLC) accounts for approximately 13-15% of all lung cancers [1, 2], and its high proliferative index is a major obstacle in overcoming this disease, even when utilizing various treatment modalities such as cytotoxic chemotherapy, radiation, and surgery. Recently, pembrolizumab, an anti-programmed death 1 (PD-1) immune checkpoint inhibitor [3], nivolumab, another anti- PD-1 antibody with or without ipilimumab, a cytotoxic T-lymphocyte antigen-4 immune checkpoint inhibitor [4], and rovalpituzumab tesirine, a delta-like protein 3-targeted antibody drug conjugate [5] demonstrated promising therapeutic outcomes in patients with SCLC. However, the majority of patients are refractory to these treatments. Thus, thorough exploration of novel treatment strategies against SCLC through a better understanding of its biology is required.

In addition, previous studies, including our report [6], have identified various clinical variables that determine the good prognosis of patients with surgically resected SCLC, such as clinical or pathological stage I and II [6, 7], female [8], lobectomy [9], and perioperative chemotherapy [8, 10]. However, the definition of those clinical variables as prognostic markers remains controversial due to the differences in the patient populations among the studies and their retrospective design. More potent and robust prognostic factors defined by molecular expression profiles are required.

Receptor tyrosine kinases (RTKs) have been reported to play a pivotal role in the biology of the proliferation and metastasis of various cancer cells [11]. However, due to the lack of opportunities to obtain sufficient tumor tissue samples by surgery, there are few well-documented studies of SCLC, that investigated the association between clinical outcomes and the expression profile of RTKs in tumors to confirm their biology.

Suppression of mediator complex subunit 12 (MED12), a subunit of the transcriptional *MEDIATOR* complex in conjunction with cell surface expression of transforming growth factor beta receptor II (TGF-βRII) has been reported to be associated with mechanisms of resistance to epidermal growth factor receptor (EGFR)-tyrosine kinase inhibitors, anaplastic lymphoma kinase (ALK) inhibitors, and chemotherapy to non-small-cell lung cancer (NSCLC) [12]. However, the expression profile of MED12 in SCLC has not yet been reported.

Based on the aforementioned findings, the primary objective of this study was therefore to identify the association between disease-specific survival and protein expression of various molecules including RTKs, MED12, and TGF-βRII by immunohistochemistry (IHC) so as to find a prognostic marker in early-stage SCLC.

## RESULTS

### Patient characteristics

**Figure 1:**
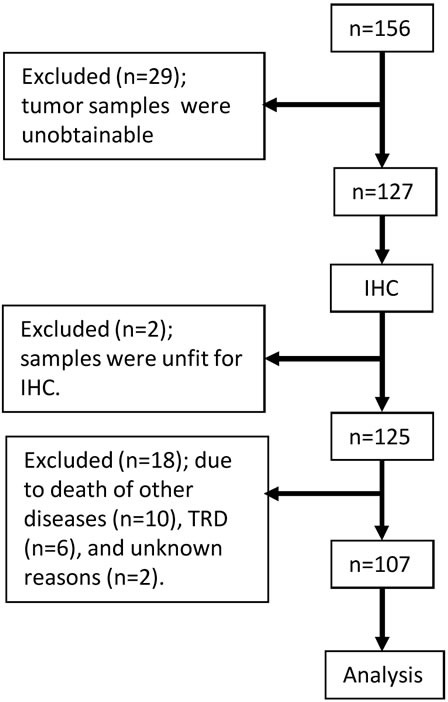
Flowchart of this study IHC; immunohistochemistry, TRD, treatment-related death.

We collected formalin-fixed paraffin-embedded (FFPE) tumor samples from 127 out of 156 patients with SCLC who underwent surgery between January 2003 and January 2013 at 16 institutions. Of the 127 samples obtained, 125 were qualitatively and quantitatively suitable for IHC. Patients who died of other diseases (*n* = 10), treatment-related causes (*n* = 6), or unknown reasons (*n* = 2) were excluded (Figure 1). The baseline characteristics of the 107 patients who were still alive at the end of the study period or had died of SCLC are listed in Table 1 and Supplementary Table S1. The median age was 70 years, 25 (23.4%) patients were female, and 9 (8.4%) were never-smokers. Thirty-three patients (30.8%) had a history or presence of other types of cancer. The numbers of patients with SCLC and combined SCLC were 76 (71.0%) and 31 (29.0%), respectively. The detailed histology which combined with SCLC was as follows: adenocarcinoma (*n* = 10), squamous cell carcinoma (*n* = 7), large cell carcinoma (*n* = 5), large cell neuroendocrine carcinoma (LCNEC) (*n* = 5), adenocarcinoma with LCNEC (*n* = 2), adenocarcinoma with squamous cell carcinoma (*n* = 1), and squamous cell carcinoma with large cell carcinoma (*n* = 1). The clinical stages (TNM version 7) were IA (*n* = 71), IB (*n* = 9), IIA (*n* = 10), IIB (*n* = 6), IIIA (*n* = 10), and IIIB (*n* = 1). The majority of the patients underwent lobectomy (72.9%) along with regional hilar-mediastinal lymph node dissection (65.4%). Adjuvant chemotherapy was conducted in 67 (62.6%) patients, including eight who received chemoradiotherapy and two who received neo-adjuvant and adjuvant chemotherapy.

**Table 1:** Demographic and clinical characteristics of patients included in this study

	Patients (n=107)	
Variables	No.	%
Age, median (range in years)	70 (52–85)	NA
Gender		
Female	25	23.4
Male	82	76.6
Smoking status		
Never-smoker	9	8.4
Smoker (current or former)	92	86.0
Unknown	6	5.6
ECOG performance status		
0	75	70.1
1	25	23.4
Unknown	7	6.5
Comorbidites or past history		
Interstitial pneumonitis	18	16.8
Other types of cancer	33	30.8
Longest tumor diameter (mm)		
Median	20	
Range	8–95	
Preoperative diagnosis of cancer		
Yes	54	50.5
No	52	48.6
Unknown	1	0.9
Histology		
SCLC	76	71.0
Combined SCLC	31	29.0
w/Ad	10	
w/Sq	7	
w/La	5	
w/LCNEC	5	
w/Ad+LCNEC	2	
w/Ad+Sq	1	
w/Sq+La	1	
Type of surgical resection		
Lobectomy	78	72.9
Segmentectomy	1	0.9
Partial resection	27	25.2
Pneumonectomy	1	0.9
Lymph node dissection		
None	25	23.4
Hilar	9	8.4
Hilar and mediastinum	70	65.4
Unknown	3	2.8
Clinical stage (TNM, version 7.0)		
IA	71	66.4
IB	9	8.4
IIA	10	9.3
IIB	6	5.6
IIIA	10	9.3
IIIB	1	0.9
Pathologic stage (TNM, version 7.0)		
IA	46	43.0
IB	19	17.8
IIA	10	9.3
IIB	4	3.7
IIIA	24	22.4
IIIB	1	0.9
IV	3	2.8
Adjuvant chemotherapy		
Yes	67	62.6
No	38	35.5
Unknown	2	1.9

SCLC, small cell lung cancer; w/, with; Ad, adenocarcinoma; Sq, squamous cell carcinoma; La, large cell carcinoma; LCNEC, large cell neuroendocrine carcinoma; NA, not applicable; ECOG, Eastern Cooperative Oncology Group; SCLC, small-cell lung cancer; TNM, tumor-node-metastasis

### Expression profile by IHC

**Figure 2:**
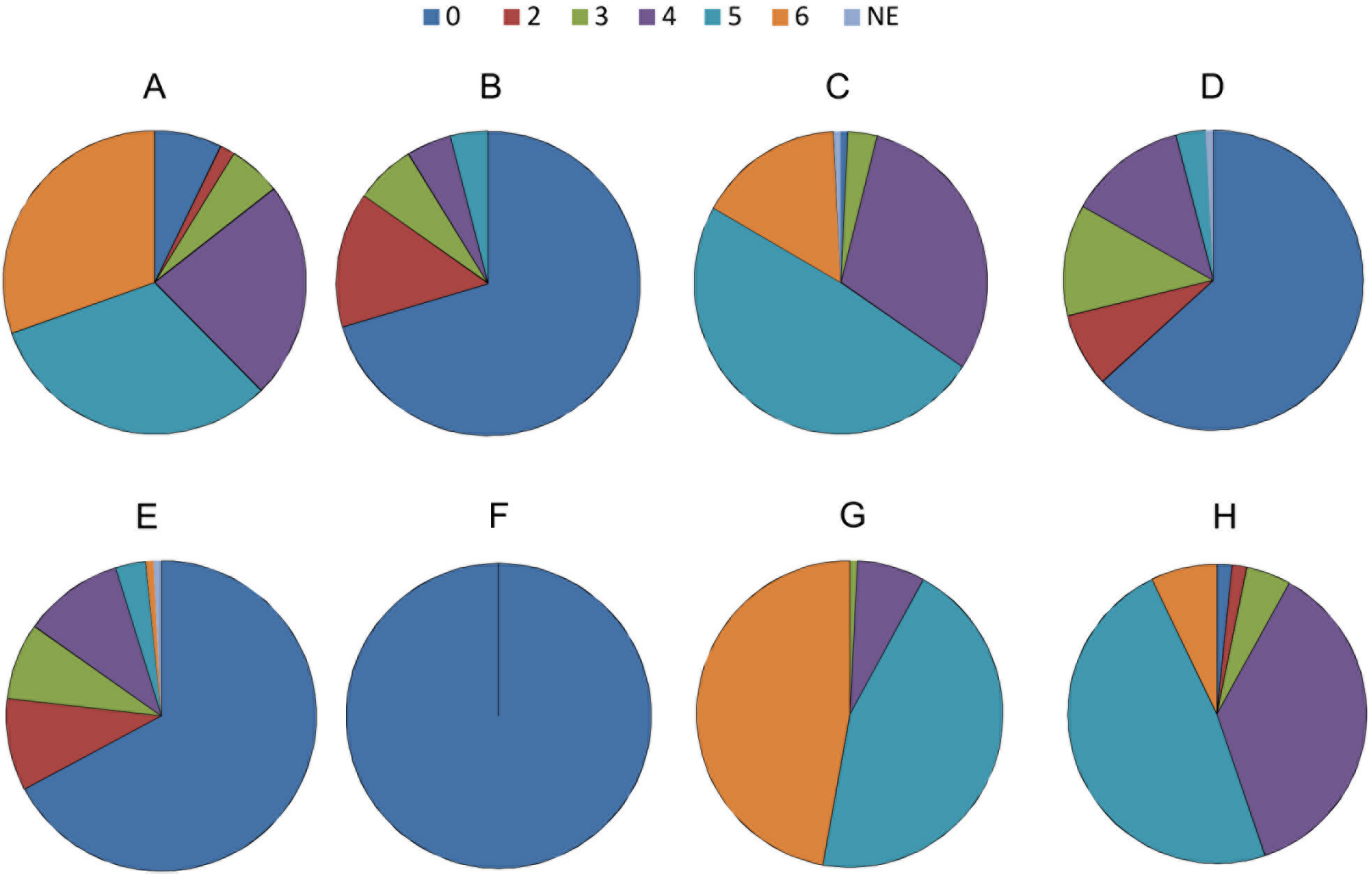
Distribution of expression level stratified by IHC total score of RTKs, MED12, and TGF-βRII **A**. c-kit, **B**. HER2, **C**. c-Met, **D**. VEGFRII, **E**. EGFR, **F**. ALK, **G**. MED12, **H**. TGF-βRII

**Figure 3:**
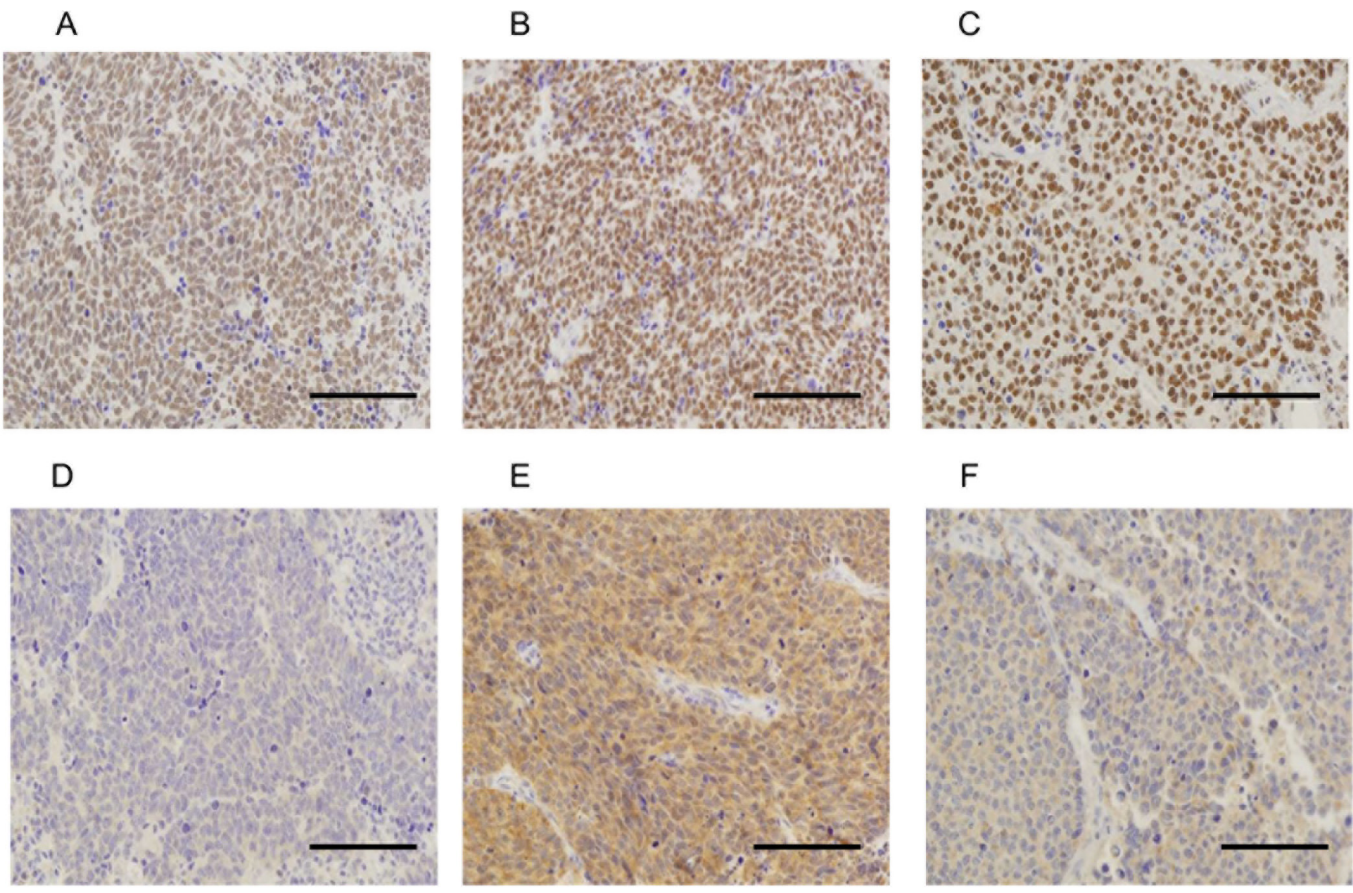
Representative examples of immunohistochemically stained sections positive for MED12 (A, B and C) and TGF-βRII (D, E and F) in tumor specimens Examples A and D, B and E, C and F are from the same patients, respectively. **A**. TS = 5, **B**. TS = 6, **C**. TS = 6, **D**. TS = 4, **E**. TS = 6, **F**. TS = 5 (×20 original magnification [A, B and C], ×40 original magnification [D, E and F]). Scale bars represent 100 μm. TS, total score = intensity score (0-3) plus proportion score (0-3) [44, 45].

Figure 2 shows the distribution of expression level stratified by IHC total score of RTKs, MED12, and TGF-βRII. The detail of IHC total score for each molecule is shown in Table 2. The majority of the samples had low expression (IHC total score [TS] = 0 to 2, refer to Materials and Methods) of EGFR (76.8%), HER2 (84.8%), and VEGFRII (71.2%), while most of the samples had high expression (TS of 5 and 6) of c-kit (62.4%), c-Met (64.8%), MED12 (92.0%), and TGF-βRII (55.2%). Of the 125 evaluable samples, none of the tumors expressed ALK. All tumors expressed MED12, and 123 samples (98.4%) expressed TGF-βRII. MED12 was highly expressed in the nucleus in 92% of the positive samples (TS = 6, refer to Materials and Methods), while TGF-βRII was highly expressed in the cytoplasm in 55% (TS = 5 and 6) of the positive samples (Figure 3). Tables 3 and 4 show the impact of various clinical variables, and that of the expression levels of RTKs, MED12, and TGF-βRII by IHC, on the overall survival (OS) of the 107 patients analyzed by univariate analysis, respectively. Among the six significant variables shown in Table 3 (*p* < 0.05), the following pairs showed a high Spearman's rank correlation coefficient (rs): i) lobectomy and hilar lymph node dissection (0.77); and ii) hilar lymph node dissection and hilar-mediastinal lymph node dissection (0.81). Thus, we excluded hilar lymph node dissection from the subsequent multivariate analysis.

**Table 2:** Distribution of RTKs, MED12, and TGF-βRII stratified by IHC total score in SCLC tumors as shown in Figure 2 (n=125)

Molecules	TS=0	TS=2	TS=3	TS=4	TS=5	TS=6	NE
c-kit	9 (7.2)	2 (1.6)	7 (5.6)	29 (23.2)	40 (32.0)	38 (30.4)	0 (0)
HER2	88 (70.4)	18 (14.4)	8 (6.4)	6 (4.8)	5 (4.0)	0 (0)	0 (0)
c-Met	1 (0.8)	0 (0)	4 (3.2)	38 (30.4)	61 (48.8)	20 (16.0)	1 (0.8)
VEGFRII	79 (63.2)	10 (8.0)	15 (12.0)	16 (12.8)	4(3.2)	0 (0)	1 (0.8)
EGFR	84 (67.2)	12 (9.6)	10 (8.0)	13 (10.4)	4 (3.2)	1 (0.8)	1 (0.8)
ALK	125 (100)	0 (0)	0 (0)	0 (0)	0 (0)	0 (0)	0 (0)
MED12	0 (0)	0 (0)	1 (0.8)	9 (7.2)	56 (44.8)	59 (47.2)	0 (0)
TGF-βRII	2 (1.6)	2 (1.6)	6 (4.8)	46 (36.8)	60 (48.0)	9 (7.2)	0 (0)

RTK, receptor tyrosine kinase; MED12, mediator complex subunit 12; TGF, transforming growth factor; IHC, immunohistochemistry; SCLC, small-cell lung cancer; TS, total score; NE, not evaluable; HER2, human EGFR-related 2; VEGFR, vascular endothelial growth factor receptor; EGFR, epidermal growth factor receptor; ALK, anaplastic lymphoma kinase. Numbers in parentheses show percentages.

**Table 3:** Univariate analysis of the association between clinical variables and OS (n=107)

Variables	HR	95% CI	*p* value
Lobectomy	0.40	0.23–0.70	0.001
c-stage I and II	0.40	0.20–0.83	0.014
Hilar lymph node dissection	0.44	0.25–0.79	0.006
Without history or presence of other types of cancer	0.47	0.27–0.81	0.007
Hilar-mediastinal lymph node dissection	0.50	0.29–0.88	0.015
Preoperative diagnosis	0.57	0.33–0.98	0.044
Adjuvant chemotherapy	0.57	0.33–1.00	0.051
Without IP complication	0.57	0.30–1.09	0.089
ECOG PS: 0	0.58	0.31–1.07	0.079
Female	0.67	0.34–1.34	0.26
Longest tumor diameter < 20 mm	0.86	0.50–1.47	0.58
Serum level of LDH < ULN	0.73	0.41–1.30	0.29
Serum level of NSE < ULN	0.83	0.35–1.96	0.67
Serum level of ProGRP < ULN	0.61	0.31–1.20	0.15
p-stage I and II	0.68	0.38–1.22	0.20
Never-smoker	0.71	0.25–1.97	0.51
Age < 70 years	0.78	0.45–1.34	0.36
Combined SCLC	0.99	0.54–1.82	0.97
VATS approach	1.08	0.62–1.87	0.80
Hospitals with high case loads	1.13	0.65–1.99	0.66

OS, overall survival; HR, hazard ratio; CI, confidence interval**;** NSE, neuron-specific enolase; ULN, upper limit of normal range; IP, interstitial pneumonitis; ProGRP, pro-gastrin-releasing peptide; ECOG PS, Eastern Cooperative Oncology Group performance status; LDH, lactate dehydrogenase; SCLC, small-cell lung cancer; VATS, video-assisted thoracoscopic surgery

**Table 4:** Univariate analysis of the association between molecular expression and OS (n=107)

Molecules	HR	95% CI	*p* value
c-kit high	0.57	0.33–0.98	0.041
HER2 high	0.64	0.27–1.50	0.30
c-Met high	1.07	0.61–1.88	0.82
VEGFRII low	0.61	0.34–1.11	0.11
EGFR low	1.11	0.52–2.36	0.79
MED12 high	1.00	0.58–1.71	1.00
TGF-βRII high	1.09	0.64–1.87	0.76

OS, overall survival; HR, hazard ratio; CI, confidence interval; HER2, human EGFR-related 2; VEGFR, vascular endothelial growth factor receptor; EGFR, epidermal growth factor receptor; MED12, mediator complex subunit 12; TGF. transforming growth factor receptor

**Figure 4:**
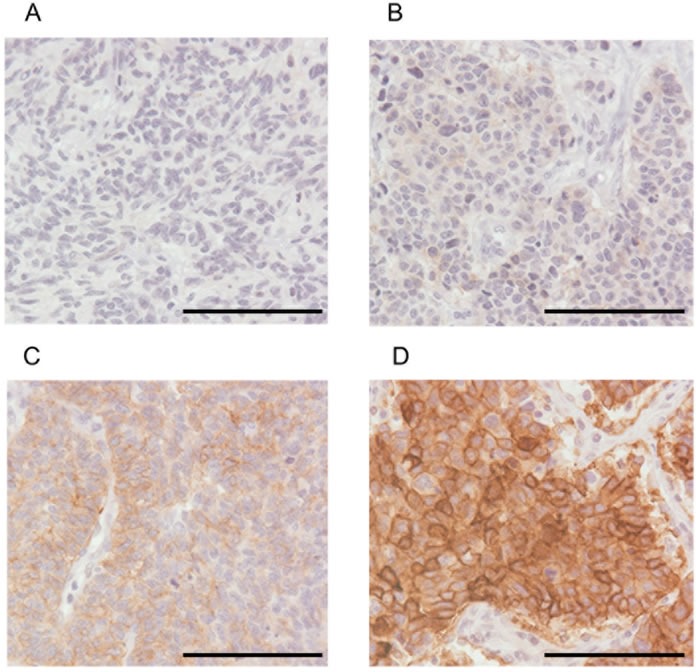
Representative examples of tumor specimens immunohistochemically stained for c-kit (×40 original magnification) **A**. TS = 0, no expression; **B**. TS = 2, low expression; **C**. TS = 4, moderate expression; **D**. TS = 6, strong expression. Scale bars represent 100 μm. TS, total score = intensity score (0-3) plus proportion score (0-3) [44, 45].

**Figure 5:**
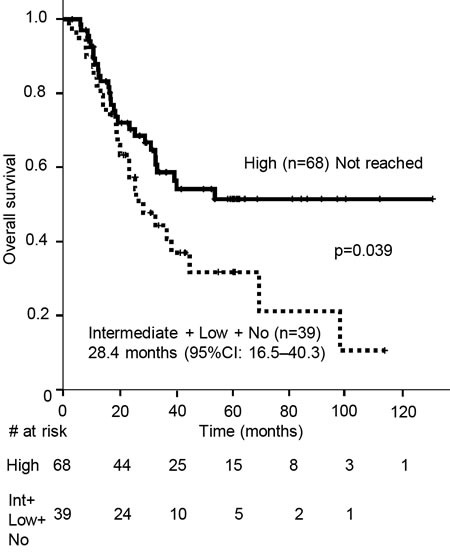
Kaplan-Meier estimates of overall survival (OS) of the patients with tumors stratified by high c-kit expression and those with intermediate, low, or moderate expression of c-kit Vertical bars indicate the censored cases at the data cutoff point. Int, intermediate; CI, confidence interval

The Kaplan-Meier estimate curves of patients stratified by the expression level of c-kit in tumors (Figure 4) are shown in Figure 5. The median follow-up time was 27.2 months (range, 2.2-130.9). The median OS of patients with high c-kit expression (TS = 5 and 6) was statistically longer than that of patients with intermediate, low, or no c-kit expression (not reached *vs* 28.4 months ([95% confidence interval (CI): 16.5-40.3], *p* = 0.039).

As shown in Table 5, multivariate analysis, including statistically significant clinical variables in the univariate analysis (Tables 3 and 4), revealed that high c-kit expression in the tumors was an independent prognostic factor (HR = 0.54, 95% CI: 0.31-0.95, *p* = 0.033).

**Table 5:** Multivariate analysis of the association between clinical variables or molecular expression and OS (n=107)

Variables	HR	95% CI	*p* value
c-stage I and II	0.26	0.12–0.57	0.001
Without history or presence of other types of cancer	0.39	0.21–0.73	0.003
Lobectomy	0.49	0.24–0.97	0.040
c-kit high	0.54	0.31–0.95	0.033

OS, overall survival; HR, hazard ratio; CI, confidence interva

**Figure 6:**
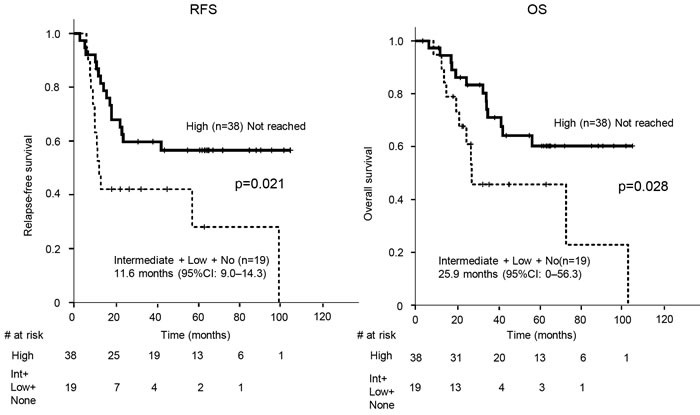
Kaplan-Meier estimates of relapse-free survival (RFS) and overall survival (OS) of the patients who underwent adjuvant chemotherapy with tumors stratified by high c-kit expression and those with intermediate, low, or no c-kit expression Vertical bars indicate the censored cases at the data cutoff point. Int, intermediate; CI, confidence interval

Next, we sought to identify the association between c-kit expression in SCLC and response to adjuvant chemotherapy, in order to address the reason for OS prolongation in patients with high c-kit expression. The relapse-free-survival (RFS) and OS of patients who underwent adjuvant chemotherapy and did not receive either neo-adjuvant chemotherapy or chemoradiotherapy (*n* = 57) were statistically longer in those with high c-kit expression (*n* = 38) than those with intermediate, low or no c-kit expression (*n* = 19) (RFS: not reached *vs* 11.6 months, *p* = 0.021; OS: not reached *vs* 25.9 months, *p* = 0.028; Figure 6).On the other hand, the RFS and OS of patients (*n* = 48) who did not receive chemotherapy (*n* = 38) or underwent other therapies, including neo-adjuvant chemotherapy (*n* = 2) and chemoradiotherapy (*n* = 8), did not show any statistical difference between patients with high c-kit expression and those with intermediate, low or no c-kit expression (RFS: *p* = 0.86; OS: *p* = 0.56, data not shown).

**Figure 7:**
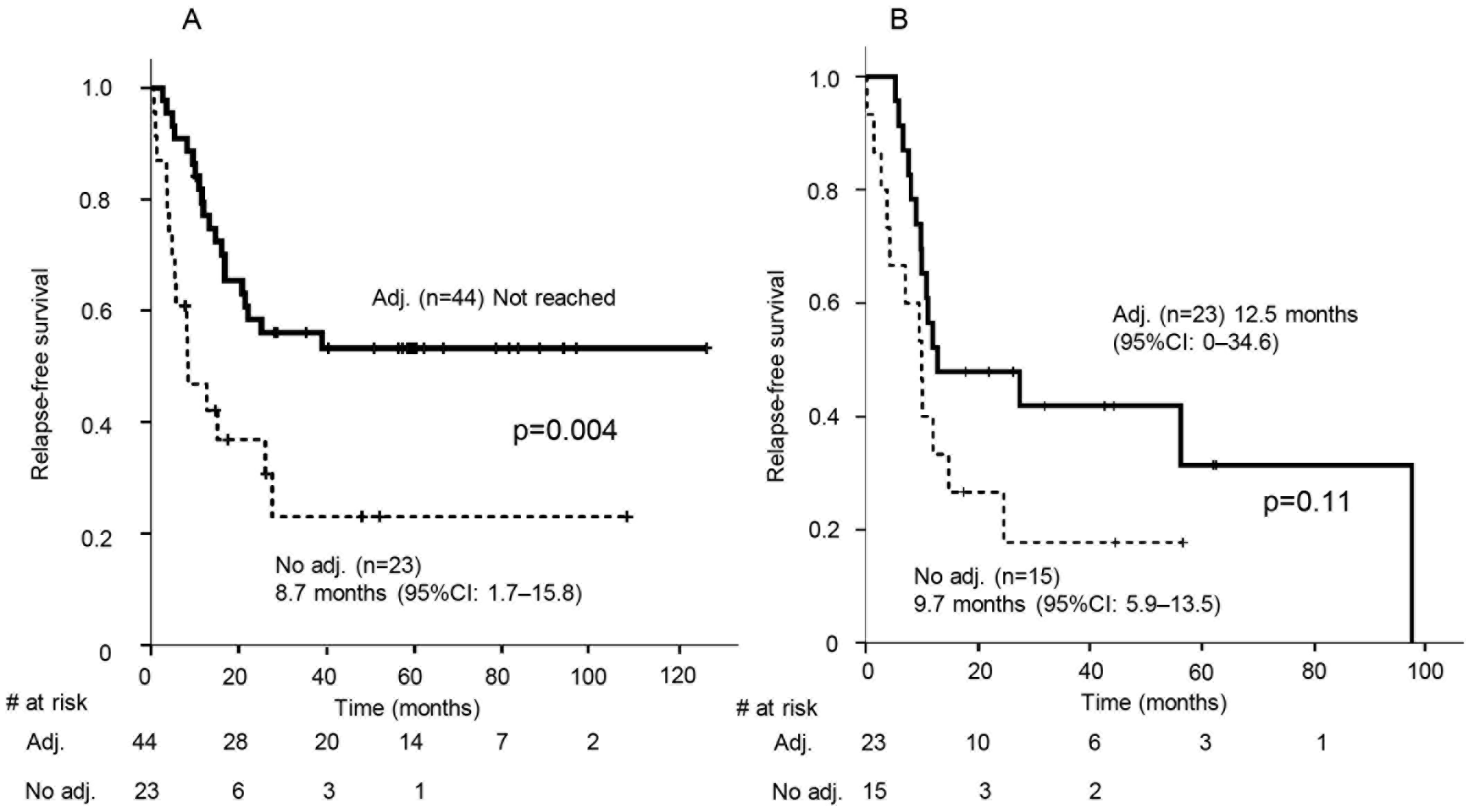
Kaplan-Meier estimates of relapse-free survival of patients with high c-kit expression (A) and those with intermediate, low, or no c-kit expression (B) with or without adjuvant chemotherapy Vertical bars indicate the censored cases at the data cutoff point. CI, confidence interval, adj., adjuvant chemotherapy

Next, we examined whether adjuvant chemotherapy was clinically meaningful to RFS prolongation in patients with high c-kit expression. The RFS of patients whose tumor had high c-kit expression was statistically longer when they underwent adjuvant chemotherapy (not reached *vs*. 8.7 months, *p* = 0.004). In contrast, the RFS of patients with intermediate, low, or no c-kit expression showed no difference between patients who underwent adjuvant chemotherapy and those who did not (12.5 months *vs*. 9.7 months, *p* = 0.11) (Figure 7).

We conducted target exon sequencing of all tumors positive for c-kit by using a next-generation sequencing system; however, no actionable mutations of *c-kit* in exons 9 or 11 were detected.

## DISCUSSION

We analyzed eight protein expression profiles of surgically resected SCLC. The results showed that high c-kit expression was a favorable prognostic marker in SCLC patients who had undergone surgical resection. The OS curve of patients with high c-kit expression and that of patients with intermediate/low/no c-kit expression separated after 20 months. The median OS of LD-SCLC patients has been reported to be approximately 20 months [13]. Patients with high c-kit expression could be a distinct subgroup of LD-SCLC, in terms of survival. Previous studies have reported conflicting evidence that high c-kit expression was either positively or negatively associated with, or had no impact on, the OS of patients with SCLC patients. These conflicting findings have also been observed in other types of cancer, for example the positive impact of high c-kit expression on gastric cancer [14] and the negative impact on NSCLC [15] and basal-like breast cancer [16]. A summary of previous reports on SCLC [17–30] is shown in Table 6. For IHC, most studies used clone A4502, a rabbit polyclonal antibody as a primary antibody for c-kit staining. The diverse association between c-kit expression and OS was presumably due to the several differences. The first difference is the type of tumor specimens, which were biopsy samples in 71.4% of the previous reports, or surgical samples (28.6%). The second difference is that of cancer stage. Twelve reports (85.7%) consisted of various combination of limited and extensive-disease SCLC, one report reviewed LD only, and another examined ED only. The third difference is varying ethnicities. Eleven studies (78.6%) were based on non-Asians, while the other 21.4% of the reports were on Asians cohorts. The fourth difference is scoring systems of positivity. A total scoring system considering both proportion score and intensity score was adopted in 28.6% of the previous reports, and only a proportion scoring system was used in 71.4%. The final difference is the variety of the cut-off values which determine positivity following the scoring systems.

**Table 6:** Previous reports demonstrating the association between c-kit expression in SCLC by IHC and OS

Author	Year	No. of samples	Specimen type	Patient population	Assoc. of OS w/ positive c-kit	Antibody (company)	Scoring system	Proportion of c-kit positive pts (%)	Ref.
Matsumura	2015	61	S	pStageI-II 79%	None*	poly (Dako)	+cell no. & intensity	48	[17]
Terada	2012	54	B	cStageI-II 63%	None	poly (Dako)	+cell no. & intensity	63	[18]
Lu	2012	23	S	pStage I-II 48%	Shorter	poly (Dako)	+cell no.	36	[19]
Erler	2011	68	S/B	NA	None	Cell Marque Corp.	+cell no.	66	[20]
López-Martin	2007	204	B#	LD 48%	None	poly (Dako)	+cell no.	73	[21]
Camps	2006	70	B (97%)	LD 46%	None	poly (Dako)	+cell no.	60	[22]
Pelosi	2004	27	S	LD 100%	None	poly (Dako)	+cell no.	5	[23]
Boldrini	2004	55	S	cStage I-II (57%##)	None	mono (DAKO & Novo)	+cell no.	18	[24]
Rohr	2004	203	B	cStage I-III 36%	Longer	poly (Dako)	+cell no.	88	[25]
Rossi	2003	27	B (82%)	LD 37%	None	poly (Dako)	+cell no. & intensity	78	[26]
Blackhall	2003	41	B	LD 69%	None	poly (Dako)	+cell no.	51	[27]
Potti	2003	193	B	ED 100%	None	poly (IMPATH)	+cell no.	28	[28]
Micke	2003	102	B (96%)	LD 30%	Shorter	poly	+cell no.	37	[29]
Naeem	2002	30	B	cStageI-II 10%	None	poly (Dako)	+cell no. & intensity	53	[30]
This study	2016	107	S	cStage I-II 90%	Longer	poly (Dako)	+cell no. & intensity	64	

IHC, immunohistochemistry; OS, overall survival; Assoc., association; pts, patients: S, surgical specimen; B, biopsy specimen; poly, polyclonal antibody; Dako, Dako Cytomation (Glostrup, Denmark); Cell Marque Corp. (Rocklin, CA, USA); Novo, Novocastra merged to Leica Biosystems (Nussloch, Germany); IMPATH, merged to A. Menarini Diagnostics (Florence, Italy); + cell no., positive cell number; NA, not available; Ref, reference. *This included large cell neuroendocrine tumor (LCNEC), comparing 3-year OS rate; # One specimen was obtained by surgery. ## n=60

The strengths of this study are as follows. First, this is the largest cohort of SCLC patients who all underwent complete resection. Surgical specimen can avoid from staining heterogeneity that is observed in biopsy samples [31]. Thus, we precisely evaluated the expression level of the molecules in the present study. Second, the present study included the highest number (20) of clinical variables available, to date. This is shown in Table 3, which also includes the precise administration rate of adjuvant chemotherapy and disease-specific survival time, not just survival time as used in most of the previous studies. The previous reports examined clinical parameters ranging from 3 [18] to 14 [21, 28]. Maximum clinical variables relevant to practice, based on a sufficient number of patients, can eventually provide precise evaluation of the association between various molecular expression and OS of the patients by univariate analysis following multivariate analysis.

The proto-oncogene *c-kit* encodes c-kit (CD117) and resides in the transmembrane domain as an RTK. Deregulation of its kinase activities has been found in the pathogenesis of cancer [32]. SCLC co-expresses c-kit and its ligand, a stem cell factor, on the surface of tumors, and this autocrine loop leads to cell proliferation [33–35].

A recent publication has demonstrated that down-regulation of c-kit in colon cancer cell lines increased expression of leucine-rich repeat-containing G-protein coupled receptor 5 and its associated genes [36], which are involved in the formation of cancer-stem/progenitor cells. SCLC tumors with little or no c-kit expression in our cohort may possess increased cancer stemness, which has been reported to lead to a reduced response to chemotherapy [37], compared to those with high c-kit expression. The results in Figure 6 showed that c-kit might be a biomarker for the determination of adjuvant chemotherapy. To validate this, we examined whether adjuvant chemotherapy was clinically meaningful in terms of RFS prolongation in patients with high c-kit expression. Figure 7 shows that tumors with high c-kit expression responded well to adjuvant chemotherapy, supporting the presence of a relationship between c-kit expression and chemosensitivity to SCLC. The regimens of all but three of the patients (94.7%) who received adjuvant chemotherapy included carboplatin- or cisplatin-based doublets (Supplementary Table S2). Thus, the expression level of c-kit in early stage SCLC tumors may determine whether platinum-based chemotherapy should be administered after surgery. Of note, there was no significant correlation between the expression levels of c-kit and either c-stage or p-stage, suggesting that, in patients undergoing adjuvant chemotherapy, the significant differences in RFS and OS between the high and low c-kit expression groups were not indicators of disease severity. These results are concordant with a previous paper by Rossi et al. [26] in which c-kit expression was down-regulated in the majority of specimens from their SCLC patients after platinum-based doublet chemotherapy. SCLC tumor clones positive for c-kit may be selectively eliminated by chemotherapy.

In the present study, adjuvant chemotherapy showed no statistical significance for OS (*p* = 0.051, Table 3) in the univariate analysis. Additionally, in the four significant variables defined by the multivariate analysis, when we excluded c-kit from the analysis and included adjuvant chemotherapy instead the significance of adjuvant chemotherapy with OS was marginal (95% CI: 0.32-1.008, *p* = 0.053). Based on our speculation with regard to the association of c-kit expression with chemosensitivity, chemotherapy could be applied more effectively to selected patients; however, this should be evaluated with an expanded cohort in a future prospective study. In addition, further investigation of the expression profiles of stem-cell markers, the c-kit pathway and its related molecules, and interactions with other tyrosine kinase signaling networks is required to clarify the reasons for the difference in the reported association between c-kit expression and OS of patients with SCLC.

To our knowledge, this is the first study to demonstrate MED12 and TGF-βRII expression in early-stage SCLC tumors. No association was observed between clinical variables including OS and the expression of MED12 and TGF-βRII (data not shown). Thus, MED12 and TGFβRII could not be a specific marker for diagnostic purposes in SCLC patients, and should not be reviewed routinely for SCLC patients.

Huang et al. [12] demonstrated that cytosolic MED12 led to the inhibition of epithelial-mesenchymal transition (EMT) through physiological contact with TGF-βRII, which conferred susceptibility of NSCLC cell lines to cytotoxic chemotherapy such as cisplatin. Our study demonstrated that MED12 expression was mainly expressed in the nucleus. Nonetheless, the sensitivity of tumors to chemotherapy can be expected from the viewpoint of the nature of treatment-naïve SCLC. The discrepancy of the MED12 location in the cell between Huang's results and ours may be as follows. Firstly, the location of MED12 expression in tumor cells might depend on the tumor origin. Shaikhibrahim et al. [38] demonstrated that MED12 expression was only observed in the nucleus in most castration-resistant prostate cancers. Secondly, mutation status might affect the MED12 location in the cell. Both SCLC and prostate cancer were reported to harbor MED12 mutations [39, 40]. These cell origins or mutation statuses prompt us to further investigate whether the activity of specific subunits influences the expression of other MEDIATOR subunits, or whether the activation of specific or bypass pathways may lead to the simultaneous upregulation of different subunits in the cytosol, which may reduce EMT-like phenotype by sequestering TGF-βRII physiologically. Thirdly, Shaikhibrahim et al. [38] also demonstrated that MED12 expression in the nucleus correlated significantly with the proliferation markers Ki67 and phosphohistone H3 (pHH3) in prostate cancer tissues, but not normal prostate tissues. They proved that MED12 expression promoted cell proliferation *in vitro* through G1-S phase transition, one of the checkpoints that platinum can target [41, 42].

Re-biopsy of the tumor that is refractory to chemotherapy is pivotal to elucidate a post-therapeutic resistance mechanism of MED12.

The limitations of this study are that it is retrospective, non-global, does not include any detailed information of preoperative staging methods, and there was a limited number of deaths (*n* = 53, 49.5%). A variety of treatment regimens were used in a heterogeneous patient population, and this could introduce another bias. In Figure 5, the numbers of censored cases under 1 year in patients with high c-kit expression and those with intermediate/low/no c-kit expression were 3 and 0, respectively. This might affect the OS difference. A large-scale prospective study using IHC for c-kit with a complete follow-up is required to investigate the prolongation of the OS of SCLC patients who undergo surgery, and to confirm our results.

In conclusion, c-kit could be a prognostic marker of patients with early-stage SCLC and this molecule should be further investigated so as to seek a potential factor in determining adjuvant chemotherapy. MED12 and TGFβRII could not be a specific marker of for diagnostic purposes in SCLC patients, and should not be reviewed routinely for SCLC patients. However, positivity of MED12 and TGFβRII in most of the tumors and the distinctive cell locations of MED12 suggest that further examination is required for the clarification of their role in SCLC. These efforts may lead to a better understanding of the biology of SCLC for developing novel treatment strategies.

## MATERIALS AND METHODS

### Patient data

Our eligibility criteria included patients with primary SCLC who had undergone complete surgical resection of a primary lung tumor between January 2003 and January 2013 at institutions participating in either the Fukushima Investigative Group for Healing Thoracic Malignancy (FIGHT) or the Hokkaido Lung Cancer Clinical Study Group Trial (HOT). Written informed consent was obtained only from patients who were still alive at the time of data accrual (from February 2013 through January 2014). The study was registered with the University Hospital Medical Information Network (UMIN) Clinical Trials Registry (identification number, UMIN000010117), and was approved by the Institutional Review Board of each participating institution. All individual data were obtained from medical records and de-identified. An unidentifiable code number was assigned to each tissue sample. Stages were determined or reclassified according to the seventh edition of the TNM staging system.

### Samples

Between January 2003 and January 2013, 156 patients were enrolled from 17 institutions. Formalin-fixed paraffin-embedded (FFPE) samples were obtained from 127 patients at 16 of those institutions (Figure 1) based on the following criteria: a complete surgical resection of primary tumors and a central re-review-confirmed pathological diagnosis of SCLC or combined SCLC according to the 2004 World Health Organization classification [43]. The FFPE tissue block was cut into 20 3-μm sections each on glass slides for IHC. The central pathological review and IHC were performed in the Department of Translational Pathology, Hokkaido University Graduate School of Medicine.

### IHC

Analyses of the association between disease-specific survival and the expression of EGFR, c-kit, human EGFR-related 2 (HER2), c-Met, vascular endothelial growth factor receptor (VEGFR) II, ALK, MED12, and TGF-βRII by IHC were performed. Rabbit anti-human polyclonal MED12 antibody (NB100-2357, Novus Biologicals, Littleton, CO, USA), rabbit anti-human polyclonal TGF-βRII antibody (NB100-91994, Novus Biologicals), rabbit anti-human polyclonal c-kit antibody (A4502, Dako, Glostrup, Denmark), rabbit anti-human polyclonal HER2 antibody (A0485, Dako), rabbit anti-human monoclonal c-Met antibody (EP1454Y, Abcam, Cambridge, UK), rabbit anti-human monoclonal VEGFRII antibody (#2479, CST, Danvers, MA, USA), mouse anti-human monoclonal EGFR antibody (31G7, Nichirei Biosciences, Tokyo, Japan), and mouse anti-human monoclonal ALK antibody (5A4, ab17127, Abcam) were used as first antibodies. Dilution of these first antibodies was 1:800 (MED12), 1:100 (TGF-βRII), 1:150 (c-kit), 1:200 (HER2), 1:150 (c-Met), 1:600 (VEGFRII), 1:50 (EGFR), and 1:50 (ALK). For antigen retrieval, slides were microwaved for 5 min four times in 1 mM of EDTA at pH 8.0. Subsequently, endogenous peroxidase activity was blocked by 3% hydrogen peroxidase in phosphate buffered saline (PBS) for 10 min. The sections were then washed in water. After blocking nonspecific binding with 10% porcine serum in PBS for 10 min, the sections were incubated with the diluted primary antibodies in a humid chamber at 4˚C overnight. After washing with water, the sections were incubated with biotinylated secondary antibodies (DAKO, 1:500) for 30 min at room temperature, washed in water again, and then incubated with peroxidase-conjugated streptavidin (DAKO, 1:500) for 30 min at room temperature. After an additional wash in water, 3, 3-diaminobenzidine was applied for 5 min, and the sections were counterstained with hematoxylin for 1 min. For MED12 staining, the nuclei of cancer cells in the specimens that showed strong immunoreactivity were used as a positive internal control. Appropriate external positive (breast cancer, according to the manufacturer's guide) and negative controls (replacing the primary antibody by nonimmune rabbit serum) were additionally evaluated.

All slides were reviewed by three pathologists without knowledge of any clinical information. The slides were scored in a method similar to that described previously [44, 45], and slightly modified from a previous report with regard to SCLC [46]. Briefly, whole fields per slide at × 200 magnification were evaluated to determine the number of stained cells and intensity. The numbers of stained cells were graded using a proportion score (PS); 0 (0% cells), 1+ (<10% cells positive), 2+ (10%-50% cells positive), or 3+ (≥50% cells positive). The intensity score (IS) was determined using a numerical scale (0: no expression; 1: weak; 2: moderate, and 3: strong expression). The TS from 0 to 6 was calculated as a sum of PS (0-3) and IS (0-3). Tumors stained by all molecules except for MED12 were divided into 4 categories: i) no expression, no tumor cells expressing cognate antigens (TS = 0); ii) low expression, (TS = 1 and 2); iii) intermediate expression (TS = 3 and 4), and iv) high expression (TS = 5 and 6). As the vast majority of tumors expressed high levels of MED12 protein, we categorized only TS = 6 into the high expression group. We analyzed the expression of all molecules by IHC in the SCLC area, not in the non-SCLC area. A specimen that shows one proportion score had at least one intensity score. Thus, there were no specimens with a total score of one.

### Targeted exon sequencing

Genomic DNA was extracted from FFPE tissues using the QIAamp DNA FFPE Tissue Kit (Qiagen, Venlo, Netherlands) in accordance with the manufacturer's protocol. The quality of genomic DNA was assessed using the Qubit dsDNA BR assay kit, the Qubit fluorometer 2.0 (Thermo Fisher Scientific, Waltham, MA, USA), and the GeneRead DNA QuantiMIZE Assay Kit (Qiagen). The GeneRead DNAseq Targeted Panel V2 (Qiagen) was used for library preparation with 59-473 ng of genomic DNA following the manufacturer's instructions. The quality of the libraries was assessed using an Agilent 2100 bioanalyzer, Agilent DNA 1000 Kit (Agilent Technologies, Santa Clara, CA, USA), and the GeneRead Library Quant Kit (Qiagen). The libraries were sequenced using MiSeq (Illumina, San Diego, CA, USA) to produce 150 bp paired-end reads. The target exon of c-kit was loaded on the TruSight Tumor Sequencing Panel (Illumina) that allows detection of major somatic mutations across 14Kb of exons (21Kb total exons and introns) in *c-kit* genes, which are commonly mutated across multiple forms of cancer. Base calling of variant frequency (VF) was performed using Miseq Reporter v2.3 (Illumina) with default parameters of VF >3.0%. The paired-end sequence reads that passed the quality control metrics determined by the pipeline were subjected to the analysis. We collected all mutation data using VariantStudio Software Tool version 2.2.4 (Illumina).

### Statistical consideration

Univariate and multivariate Cox proportional hazard model analyses were performed to examine the association between disease-specific survival of 107 patients and protein expression of RTKs, MED12, and TGF-βRII in the tumor samples from the patients. For clinical variables that were significant in the univariate analysis, we confirmed the rs and excluded multiple variables with a high correlation (rs ≥0.6) and similar significance in the subsequent multivariate analysis. Survival time was calculated from the date of surgery until the date of death from any cause. Patients who had not survived through the observation period were censored at the last available information on status. Survival curves were drawn by the Kaplan-Meier method and statistically compared using the log-rank test. All statistical analyses were performed using SPSS version 20 (IBM Corporation, Armonk, NY, USA). A *P* value of <0.05 was considered to be statistically significant.

## SUPPLEMENTARY TABLES


