# Supplementary material for: Immunohistochemical profiling of receptor tyrosine kinases, MED12, and TGF-βRII of surgically resected small cell lung cancer, and the potential of c-kit as a prognostic marker
Source: Oncotarget. 2016 Dec 31;8(24):39711–26. doi: 10.18632/oncotarget.14410 (PMC5503646; doi:10.18632/oncotarget.14410)
Supplement: Supplementary file 1 [file oncotarget-08-39711-s001.pdf]

Immunohistochemical profiling of receptor tyrosine kinases, MED12, and TGF-βRII of surgically resected small cell lung cancer, and the potential of c-kit as a prognostic marker

Supplementary Material

Supplementary Table S1. Additional patient characteristics

	Patients (n=107)	
	No.	%
Serum level of LDH		
< ULN	75	70.1
≥ ULN	30	28.0
Unknown	2	1.9
Serum level of ProGRP		
< ULN	67	62.6
≥ ULN	20	18.7
Unknown	20	18.7
Serum level of NSE		
< ULN	50	46.7
≥ ULN	17	15.9
Unknown	40	37.4
Approach		
VATS	66	61.7
Open surgery	41	38.3
Perioperative chemoradiotherapy		
Yes	9	8.4
No	96	89.7
Unknown	2	1.9
Salvage surgery		
Yes	1	0.9
No	105	98.1
Unknown	1	0.9
PCI		
Yes	8	7.5
No	97	90.7
Unknown	2	1.9

LDH, lactate dehydrogenase; ULN, upper limit of normal range; ProGRP, pro-gastrin-releasing peptide; NSE, neuron specific enolase; VATS, video-assisted thoracoscopic surgery; PCI, prophylactic cranial irradiation

Supplementary Table S2. Adjuvant chemotherapy regimens

	Patients (n=57)	
	No.	%
Platinum-based doublet	54	94.7
CBDCA + VP-16	22	38.6
CDDP + VP-16	18	31.6
CDDP + CPT-11	10	17.5
CBDCA + CPT-11	4	7.0
AMR	1	1.8
S-1	2	3.5

CBDCA, carboplatin; VP-16, etoposide; CDDP, cisplatin; CPT-11, irinotecan; AMR, amrubicin
